# Patient-derived pancreatic cancer-on-a-chip recapitulates the tumor microenvironment

**DOI:** 10.1038/s41378-022-00370-6

**Published:** 2022-03-31

**Authors:** Muhammad R. Haque, Caitlin R. Wessel, Daniel D. Leary, Chengyao Wang, Abhinav Bhushan, Faraz Bishehsari

**Affiliations:** 1 Division of Digestive Diseases, Rush Center for Integrated Microbiome & Chronobiology Research, Rush University Medical Center, Chicago, IL 60612 USA; 2 University of Louisville School of Medicine, Louisville, KY 40202 USA; 3 Department of Biomedical Engineering, Illinois Institute of Technology, Chicago, IL 60616 USA

**Keywords:** Materials science, Engineering

## Abstract

The patient population suffering from pancreatic ductal adenocarcinoma (PDAC) presents, as a whole, with a high degree of molecular tumor heterogeneity. The heterogeneity of PDAC tumor composition has complicated treatment and stalled success in clinical trials. Current in vitro techniques insufficiently replicate the intricate stromal components of PDAC tumor microenvironments (TMEs) and fail to model a given tumor’s unique genetic phenotype. The development of patient-derived organoids (PDOs) has opened the door for improved personalized medicine since PDOs are derived directly from patient tumors, thus preserving the tumors’ unique behaviors and genetic phenotypes. This study developed a tumor-chip device engineered to mimic the PDAC TME by incorporating PDOs and stromal cells, specifically pancreatic stellate cells and macrophages. Establishing PDOs in a multicellular microfluidic chip device prolongs cellular function and longevity and successfully establishes a complex organotypic tumor environment that incorporates desmoplastic stroma and immune cells. When primary cancer cells in monoculture were subjected to stroma-depleting agents, there was no effect on cancer cell viability. However, targeting stroma in our tumor-chip model resulted in a significant increase in the chemotherapy effect on cancer cells, thus validating the use of this tumor-chip device for drug testing.

## Introduction

Pancreatic ductal adenocarcinoma (PDAC) accounts for 93% of cancers arising from the pancreas^1^. PDAC is considered the most fatal of all cancers and is associated with an abysmal prognosis. It is anticipated to be the second leading cause of cancer-related death in the USA by 2040^2,3^. The survival rate has only increased from 3 to 10% in the last five decades, and surgery remains the only possible curative strategy^3^. Surgical resection combined with chemoradiation may improve the survival rate; however, it is still not above 20–25%^4^. FOLFIRINOX, a combination of fluorouracil, leucovorin, irinotecan, and oxaliplatin, has been used to treat metastatic PDAC patients, although this method has not shown ideal outcomes^5^. Unfortunately, improved FOLFIRINOX with gemcitabine as an adjuvant did not increase survival rates^6^. These challenges suggest that there is an urgent need to identify effective treatments for combating this deadly cancer.

Multiple targeted therapies with promising preclinical profiles have emerged over the past decade, but no drug has been shown to exert an expected response in the setting of a clinical trial. The lack of a remarkable clinical response is partly due to the large molecular heterogeneity among PDAC patients; consequently, only a small subset of patients who may potentially benefit from these drugs are included in clinical trials. Instead, a personalized approach (precision medicine, PM) that uses tumor-related information to predict the drug response can be used.

Advances in three-dimensional (3D) culture techniques have enabled the formation of organoids from patient tissues, bringing us closer to individualized PM in pancreatic cancer^7^. Organoids are progenitor cells that possess the phenotype of the tissue of origin. Unlike primary cells, organoids can be propagated long-term ex vivo, allowing for the study of patient-specific cancer readouts. Tissue-derived stem cells can self-organize themselves into sphere-shaped organoids composed predominantly of epithelial cells^8^. Pancreatic organoids can be developed from pancreatic tissue samples obtained via fine-needle biopsy (FNB), and patient-derived organoids (PDOs) from PDAC could help advance PM in PDAC^9^. However, PDOs are mainly epithelial and lack the tumor microenvironment (TME) in PDAC, which is characterized by the infiltration of immune cells and fibroblasts.

Stromal cells and cancerous epithelial cells have bidirectional crosstalk that creates a desmoplastic stroma within the microenvironment, thus promoting tumor progression and chemoresistance^10–12^. Tumor-associated macrophages (TAMs) are one of the major immune cell types in the TME. On the other hand, cancer-associated fibroblasts (CAFs) are the primary source of extracellular matrix deposition. Substantial evidence has suggested the notorious role of these components in cancer progression, metastasis, and chemoresistance^7,13^. Although developing an in vitro system that can faithfully recapitulate the PDAC TME is technically challenging, our goal is to advance the current PDO technology by establishing a tumor-chip platform, including PDOs and major stromal components (i.e., fibroblasts and macrophages), to better mimic the in vivo PDAC environment.

Microfluidic chips are novel devices that contain chambers for cells to grow while simultaneously allowing for the constant perfusion of cell culture medium. Such a device has enormous potential applications, from recapitulating complex 3D TMEs to testing anticancer drugs. Moreover, it can be an excellent tool for making a PM-based platform and transitioning research from bench to bedside by providing a drug screening platform prior to clinical application^7^. In this study, we established an orthotopic PDO-based chip model of PDAC in which bidirectional epithelium-stroma interactions were present. We then functionally tested our model by examining the enhancing effect of microenvironment-modulating agents on the antitumor efficacy of chemotherapy in our tumor-chip model.

## Results

### Bidirectional interactions between neoplastic pancreatic organoids and stromal cells promote synergistic growth

**Fig. 1:**
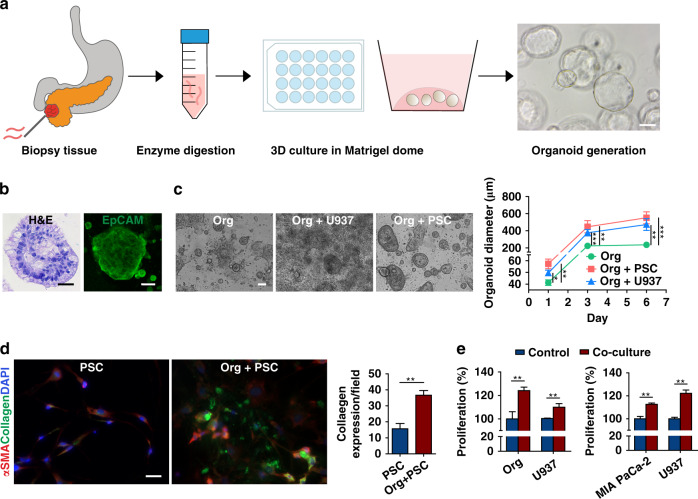
Patient-derived organoid (PDOs) propagation, characterization, and bidirectional interactions with stromal cells in welled plates. **a** Schematic describing the workflow used to generate PDOs from biopsy samples via fine-needle biopsy (FNB). PDOs (generated from the biopsy sample of one patient) formed sphere-shaped cell clusters in 3D Matrigel culture. Scale bar, 100 µm. **b** Characterization of the isolated PDOs by H&E and EpCAM staining. Scale bar, 100 µm. **c** PDOs cocultured with PSCs and U937 monocytes showed an increased average diameter (±SEM, *n* = 3) over a 6 day culture period in welled plates. Scale bar, 100 µm. **d** Coculture of the organoids with PSCs significantly increased collagen deposition (*n* = 3 wells, expression quantified from ten fields). Scale bar, 20 µm. **E** Bidirectional increase in the proliferation of neoplastic epithelial cells (PDO and MIA PaCa-2) and U937 monocytes in a transwell culture assessed by a cell-counting kit assay (*n* = 5, CCK8). *p < 0.05, **p < 0.01, ***p < 0.001

Biopsy samples from PDAC patients were collected using ultrasound-guided FNB (Fig. 1a). The isolated organoids were developed and cultured in 24-well plates for two passages before conducting experiments. The organoids continued to propagate with each passage, giving rise to new spherical organoids in the 3D Matrigel environment. H&E staining highlighted the complex structure of the organoids with densely packed cell clusters (Fig. 1b). Similar to the tissue of origin, the organoids expressed abundant epithelial cell adhesion molecule (EpCAM), a tumor epithelial transmembrane protein^14^.

The mean organoid diameter in the stroma (PSC and U937) cell coculture started to increase from Day 1 and continued to grow through Day 6, reaching a diameter significantly greater than that of organoids cultured alone (Fig. 1c). PSCs secreted more collagen (*p* < 0.01, org vs. org + PSCs) in the presence of organoids, as observed via immunofluorescence imaging (Fig. 1d). Coculture of organoids and U937 cells induced the proliferation of both cell types (*p* < 0.01*)* compared with monoculture (Fig. 1e, left). Bidirectional proliferative effects of cancer cells and U937 cells were also observed in MIA PaCa-2 cells, a pancreatic cell line (Fig. 1e, right). We observed significantly increased (*p* < 0.01*)* proliferation of MIA PaCa-2 and U937 cells in the coculture compared with that in the monoculture.

**Fig. 2:**
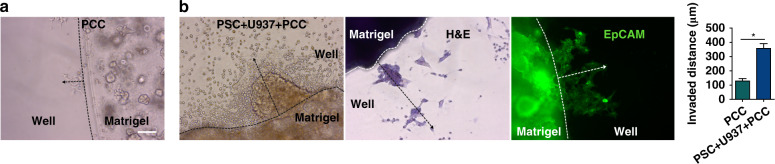
Stromal cells increase the invasiveness of cancerous epithelial cells (PDOs). Invasion assay was performed by coculturing PDOs with stromal cells in Matrigel in welled plates (*n* = 3 wells/group). **a** Monocultured primary cancer cells (PCCs) were used as a control (*n* = 3 wells). **b** After 6 days of coculture, the PCCs traveled a significantly longer distance, indicating induced invasiveness of the PCCs by the stromal cells. H&E and EpCAM staining confirmed the invaded cells as PCCs. The traveled distance was measured from the edge of the Matrigel dome along the dotted arrow. The bar graph shows the mean invaded distance (µm) ± SEM, *p < 0.0001. Scale bar, 100 µm

To observe whether stromal cells can induce invasiveness in primary cancer cells (PCCs, interchangeably used instead of organoids), we performed an invasion assay by culturing the cells in Matrigel (Fig. 2). As observed, the PCCs sprouted out of the Matrigel matrix and migrated, with the distance of migration measured at the time of imaging on Day 6 (Fig. 2a). The distance traveled by the PCCs in coculture with the stromal cells was greater (*p* < 0.0001 vs. PCC monoculture), suggesting induced invasiveness of the PCCs in coculture. H&E and EpCAM staining confirmed that the migrated cells were PCCs (Fig. 2b).

### Culture of PCCs on a microfluidic chip

**Fig. 3:**
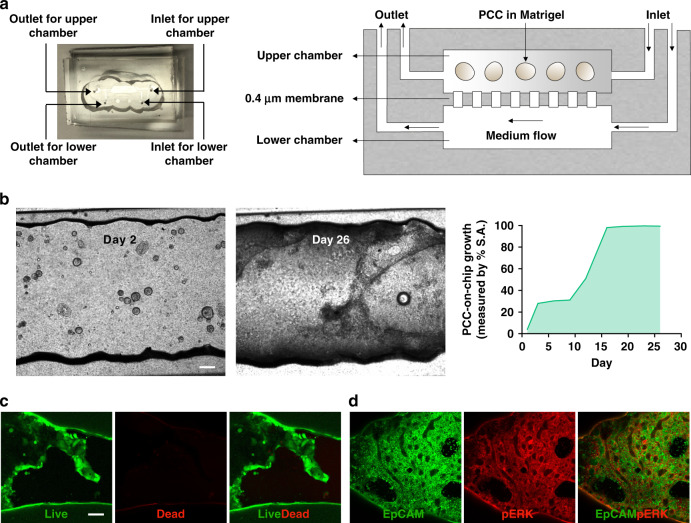
A two-chamber microfluidic chip for the seeding and growing of PDOs. **a** Image and schematic of the multichamber microfluidic device. The chip consists of two chambers separated by a 0.4 µm porous membrane. Cells are installed in the upper chamber through the inlet. Perfusion of the cell culture medium through the lower chamber maintains cell viability. **b** Top view images of the cell-laden chip. PDOs were cultured for 26 days with continuous perfusion of the medium. After 1 week, the organoids lost their 3D spherical shapes and spread two-dimensionally to occupy the inner surface area (S.A.) of the chip (right panel). On Day 26, **c** viability and **d** pERK expression were assessed in the PCCs to determine survival in the organ-chip device. Scale bar, 200 µm

Our microfluidic chip consists of two chambers separated by a porous membrane (Fig. 3a). The inlet to the upper chamber serves as the access point for installing cells into the upper chamber. The inlet to the lower chamber is connected with a syringe containing media (organoid medium: RPMI = 1:1) via a tube. The syringe is placed on a pump for continuous perfusion of the medium at 5 µL/h throughout the experimental period. To observe the growth of PCCs, 2000 organoids suspended in 50% Matrigel were loaded in the upper chamber of each chip, followed by a 26-day culture (Fig. 3b). After 1 week, the organoids transformed from a spherical shape into a two-dimensional (2D) structure. These cells are referred to as PCCs and spread throughout the chamber. The PCCs occupied the whole inner space of the chamber within 16 days. 3D cultures are essential to study cell behavior since they recapitulate the tumor microenvironment in vivo^15^. Matrigel is a soluble basement membrane extract that forms a gel at a physiological temperature. We used Matrigel because of its vast publication record as a standard ECM-based scaffold for cancer cell culture. However, unlike alginate hydrogels, where structural stiffness can be controlled, Matrigel lacks mechanical robustness and degrades rapidly in culture due to the migratory activity of cancer cells^16^. Initially, the organoids were supported by Matrigel to retain the 3D spherical structure in the chips, but the eventual transformation into a 2D layer may have been a result of Matrigel degradation.

We performed a live-dead viability assay to confirm whether the 2D cell layer on the chip surface was viable. As indicated by green fluorescence, the PCCs were viable with limited red staining of dead cells. Moreover, the EpCAM-expressing cancer cells also showed positive staining for pERK, a KRAS downstream effector in PDAC (Fig. 3c, d)^14,17^. The expression levels of EpCAM and pERK in the cancerous epithelial cell membrane indicate cell survival and growth. Several fluorescence images were obtained throughout the chamber. However, the representative live-dead viability images (Fig. 3c) were captured near the inlet and the expression of the cell surface markers (Fig. 3d) was obtained near the outlet of the upper chamber. The viability of the cells was unchanged regardless of their location in the channel because of the continuous cell culture medium perfusion through the lower channel during the experimental period. However, we performed coculture and drug treatment studies within 9 days of culture in subsequent experiments, as there were still 3D organoids present.

### Recapitulating the PDAC tumor microenvironment on a chip

**Fig. 4:**
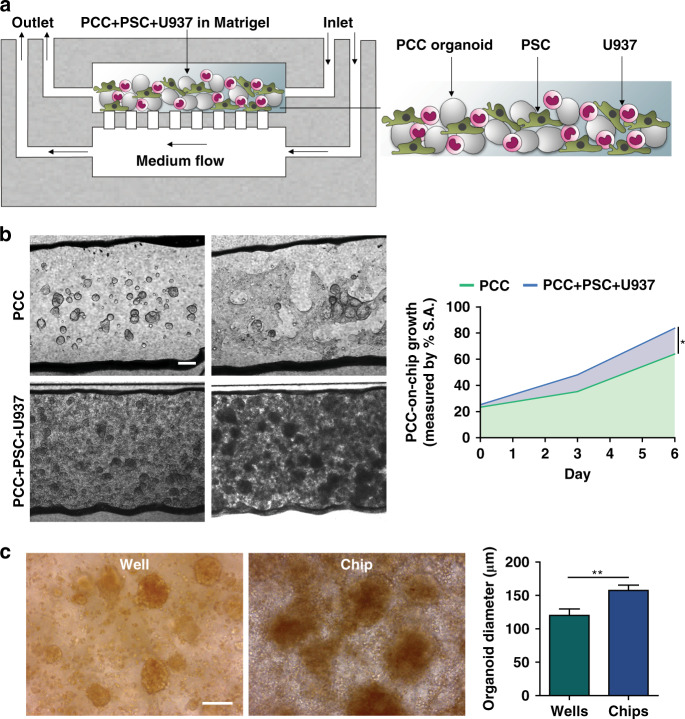
Growth of the primary cancer cells (PCC) with stromal cells in chips. **a** Schematic of PCC + stromal cell seeding on the microfluidic device (*n* = 3 chips). **b** PCCs grew to significantly higher density when cocultured with stromal cells and occupied more space than PCCs in PCC monoculture in chips, indicating ongoing bidirectional proliferation. Scale bar, 200 µm. The area-filled graph shows the PCC growth dynamics on the chip’s inner surface area (S.A.). **c** Organoids that grew on a chip had a significantly greater diameter than the organoids grown in a welled plate. Images were acquired from organoids cultured in three wells/chips and quantified from ten fields using ImageJ software to determine the average organoid diameter ± SEM. Scale bar, 100 µm. *p < 0.05, **p < 0.01

**Fig. 5:**
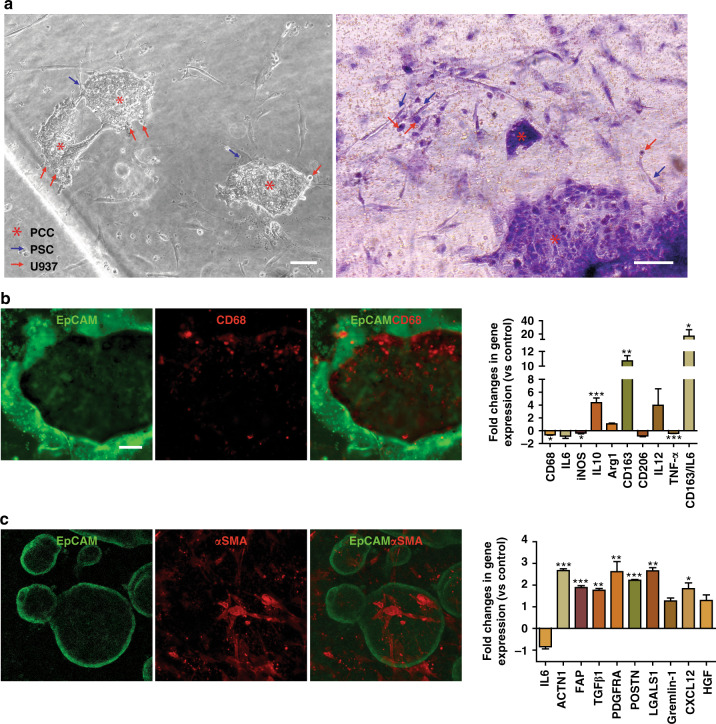
Recapitulation of the PDAC TME in chips. **a** Phase-contrast and H&E staining assessment of the cell-laden chips after 6 days of coculture with continuous medium perfusion. **b**, **c** Immunostaining of PCCs for EpCAM, U937 cells for CD68, and PSCs for α-SMA. The expression profile of human U937 monocytes and PSCs in the organoids was performed by qPCR analysis. The fold change in gene expression was normalized to the housekeeping gene GAPDH and compared to the cells in monoculture. Data were expressed as the mean ± SEM, *n* = 3 per experiment. Scale bar, 100 µm. *p < 0.05, **p < 0.01, ***p < 0.001

To recapitulate the features of the PDAC TME, we incorporated stromal cells with PCCs and loaded them into the upper chamber of the chip (Fig. 4a). Six days was sufficient for PCC growth and occurrence of the desmoplastic reaction (Fig. 4b). The PCCs grew significantly faster in the stromal cell coculture chip and occupied 83.6% of the chamber space compared with 63.9% in the monoculture (*p* < 0.05) by Day 6, confirming the influence of stroma on PCC proliferation in the chip. Compared to the chip, a parallel welled plate coculture of the cells demonstrated significantly lower (*p* < 0.01) cancer cell growth, which further supports the use of the chip as a superior platform to recapitulate the vigorous growth of cancer cells (Fig. 4c). Bright-field imaging and H&E staining showed the intimate interaction of the PCCs with the stromal cells in the chip (Fig. 5a). Staining for CD68 and α-smooth muscle actin (α-SMA) in U937 cells and PSCs, respectively, revealed the presence of stromal cells around epithelial PCCs (Fig. 5b, c, left).

We further characterized PSCs and U937 cells in coculture at the gene expression level using several markers. Macrophages showed increased expression of several protumorigenic (i.e., M2-type) markers in the coculture (M2/M1: CD163:IL6, *p* < 0.05, Fig. 5b, right)^18^. M2/M1 polarization was calculated as the average fold change in M2 gene (CD163) expression over that in M1 gene (IL 6) expression. The reduced population of CD68+ cells in the cocultured U937 monocytes explains the high shift toward the M2 phenotype. Nonetheless, PSC marker profiling revealed significantly higher expression of cancer-related genes (ACTN1, *p* < 0.001; FAP, *p* < 0.001; TGFβ1, *p* < 0.01*;* PDGFRA, *p* < *0.01;* POSTN, *p* < *0.001;* LGALS, *p* < 0.01*;* CXCL12, *p* < 0.05), which is in line with previous reports (Fig. 5c, right)^19^.

### Anti-stroma agents augment the ex vivo chemotherapy response in PCC

**Fig. 6:**
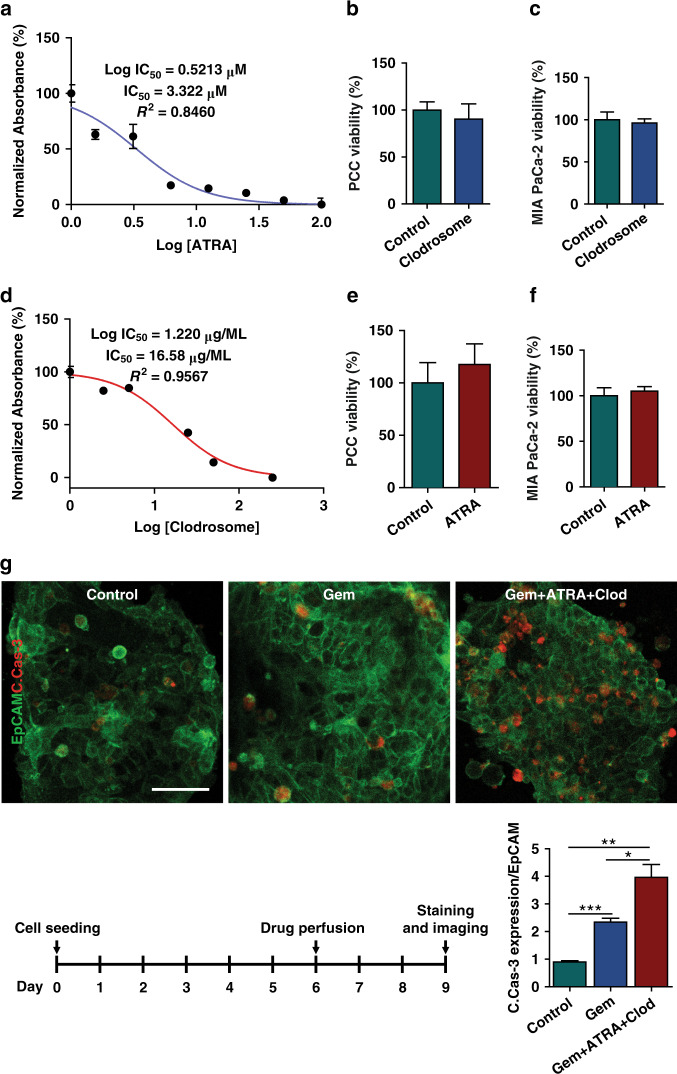
Effect of stroma depletion in chemotherapeutic response. To show the utility of the multicellular PDO-based organ-chip system for drug screening, stroma-depleting agents were used along with gemcitabine. The IC_50_ values of **a** Clodrosome® (liposomal clodronate) and **d** ATRA were measured in U937 cells and PSCs, respectively, using a CCK8 assay. **b**, **c**, **e**, **f** The IC_50_ values of the drugs were confirmed to be noncytotoxic to the PCCs and MIA PaCa-2 cells, as observed in the viability assay. **g** PDOs and stromal cells were grown on the organ-chip device for 6 days, followed by drug perfusion for 3 days. Immunofluorescence staining demonstrated the augmented effect of gemcitabine in combination with stroma-depleting agents compared with gemcitabine alone. ImageJ^®^ software was used to calculate the intensity of cleaved caspase-3 (C.Cas-3, red) in EpCAM (green)-positive cells. Scale bar, 50 µm. *p < 0.05, **p < 0.01, ***p < 0.001

To functionally characterize our chip model for drug response testing, we hypothesized that targeting PSCs (by ATRA) or macrophages (by Clodrosome^®^) combined with chemotherapy would be more effective than chemotherapy alone. We first determined the IC_50_ values of ATRA, Clodrosome^®^, and gemcitabine in PSCs, U937 cells, and organoids, respectively (Fig. 6a, d and Fig. S1). The stroma-depleting agents at their IC_50_ values did not affect the viability of PCCs or MIA PaCa-2 cells (Fig. 6b, c, e, f).

Organoids from one patient were used to check the ex vivo drug response of the PCCs. The cells were inoculated into the chips with stromal cells and cultured for 6 days. On Day 6, either gemcitabine or a combination of gemcitabine with ATRA and Clodrosome^®^ at their IC_50_ values were perfused into the chips for 72 h followed by PFA fixation and staining on Day 9 (Fig. 6g). Compared to no treatment, gemcitabine-induced significant PCC apoptosis as assessed by PCC expression of cleaved caspase-3 (C.Cas-3-positive in EpCAM-positive cells, *n* = 5 chips/group). The incorporation of stroma-depleting agents further increased apoptosis by almost 2-fold at a statistically significant rate (*p* < 0.05 vs. gemcitabine treatment). PCC viability and survival in the chip were previously confirmed in a 26-day culture by live-dead staining and the coexpression of the cell surface markers EpCAM and pERK (Fig. 3c, d). Therefore, the observed C.Cas-3 expression in EpCAM-positive cells was undoubtedly from a drug effect rather than cellular decay in the culture system. The results show the enhancing effects of TME-modulating agents on the antitumor efficacy of chemotherapy in our tumor-chip model.

## Discussion

This study utilized a tumor-chip device to recapitulate the TME of PDAC using PDOs and stromal cells. Such a device could be implemented as an ex vivo PM-based drug testing platform prior to the onset of clinical treatment. We studied PDAC since it has one of the worst prognoses and survival rates^1,3^. Due to the noteworthy molecular heterogeneity among PDAC patients, there is a dire need for a PM-based treatment option. Here, using a microfluidic chip device, we cocultured stromal cells with primary cancer organoids to recapitulate the TME and observed active crosstalk between cancer and stromal cells ex vivo. As a proof of concept study, we showed that targeting the stroma augmented the chemotherapy effect in our system without directly killing the cancer cells.

Patient-derived organoids have been shown to recapitulate patient clinical responses to targeted therapies, highlighting the potential of PDOs as critical research tools that aid in identifying subpopulations that respond to PM-based therapy^20^. However, organoid cultures mainly contain ductal epithelial cells and do not resemble the microenvironment of PDAC tumors. Therefore, it is crucial to include the key cell types involved in the TME to create PDAC-on-a-chip devices.

The PDAC TME has a strong influence on disease progression, drug response, and immune evasion via interactions between cancerous epithelial cells and stromal cells^7^. Stromal cells, such as PSCs, secrete extracellular matrix (ECM) proteins that create a dense stroma, referred to as “desmoplasia,” within the TME. As reported widely in various cancer types, PSCs can impart chemoresistance and reduce cancer cell death by releasing soluble factors or increasing cancer cell stemness^21,22^. Our study confirmed the bidirectional effects between PSCs and primary cancer cells, as organoid diameter increased upon coculture (Fig. 1c). At the same time, collagen secreted by the cocultured PSCs was significantly elevated compared to that secreted by the cells in the monocultures (Fig. 1d). It has been reported that activated fibroblasts produce excessive ECM components by adopting a myofibroblast phenotype^23^. In our qPCR analysis, we observed that PSCs cocultured with organoids expressed significantly higher (*p* < *0.001*) levels of alpha-smooth muscle actin (α-SMA, encoded by ACTN1) and fibroblast activation protein-alpha (FAP), which are markers of PSC activation (Fig. 5c)^24,25^. Moreover, the activated PSCs expressed growth factors, inflammatory cytokines, and chemokines associated with tumor growth and metastasis, which helped the neoplastic organoids proliferate (Figs. 1b, 5c). This symbiotic relationship between fibroblasts and cancer cells is similar to that in a previous report^23^.

Among infiltrating immune cells in the TME, macrophages play a crucial role in tumor growth, progression, and chemoresistance^26,27^. Previously, using mouse cells, we successfully implemented an organoid-macrophage coculture system in Transwell plates that helped us study the dynamic interaction between these cells^28^. We found bidirectional signaling initiated by epithelial KRAS that promoted protumorigenic expression patterns in macrophages, which in turn amplified cancerous phenotypes in the epithelium. Here, using human primary pancreatic cancer cells and the MIA PaCa-2 cell line, we observed increased proliferation of cancerous epithelial cells and monocytes upon coculture (Fig. 1e).

During metastasis, cancer cells undergo an ‘invasion’ step that involves cell movement within tissues before spreading into the circulation^29^. Although CAFs have been shown to have protective effects^30,31^, most of the well-accepted studies agree with their role in promoting cancer cell invasion^32–35^. The cytokines, chemokines, and growth factors secreted by CAFs create a favorable TME by remodeling the ECM, which eventually facilitates invasion^34^. Furthermore, CAFs can directly exert cancer invasion through epithelial to mesenchymal transition (EMT) by expressing N- and E-cadherin^36^. TAM-induced inflammation is also responsible for causing EMT^13^. TAMs are the most abundant immune cell type in the PDAC TME^13^. As the tumor progresses, TAMs change their phenotype from M1 to M2, participating in tumor cell migration and invasion. By adopting a widely used in vitro invasion assay using Matrigel, we confirmed the increased invasiveness of the cancerous epithelium in coculture with stromal cells (Fig. 2)^37–39^. Concomitantly, direct contact between cells also leads to ECM remodeling and invasion of cancer cells, which is consistent with existing reports^19,34^. Therefore, to establish a pancreatic cancer-on-a-chip model, we argue that it is essential to include these stromal cells to better mimic the PDAC TME.

Given the critical role of the PDAC TME in tumor growth and drug response, microfluidic chips can be a promising tool because they can be used to construct individualized tumor chips for a given patient’s cancer^7^. The tumor-chip devices provide multiple compartments to culture-specific cell types in specific chambers, unlike traditional welled plates. Moreover, tumor-chip devices can be constantly perfused with cell-culture medium, thereby providing a steady supply of nutrients and oxygen to the cultured cells. The primary cancer cells survived for a long time on the chips and expressed PDAC markers (Fig. 3c, d). Cancer organoids in coculture grew more on the chips than in their welled plate counterparts, affirming the superiority of this platform in recapitulating tumor growth (Fig. 4c).

Several tumor-chip models have been developed to recapitulate the pancreatic cancer TME^23,40–43^. Our model is the first to incorporate patient-derived primary cancer cells with two relevant human stromal cells (fibroblasts/macrophages) in PDAC (Fig. 4). The neoplastic epithelial cells caused the stromal cells to transition toward pro-cancerous phenotypes, as observed by changes in gene expression levels (Fig. 5). Using our tumor-chip model, we demonstrated the adjuvant effects of anti-stroma agents in PDAC as a proof of concept for using our multicellular patient-derived organoid-based system for drug screening. To assess microenvironment-modulating agents, we targeted PSCs with all-trans retinoic acid (ATRA), which induces PSC quiescence and has been shown to reduce the proliferation of surrounding pancreatic cancer cells^44^. Recently, a phase I study was conducted in which ATRA was shown to augment the effects of gemcitabine in patients with advanced PDAC^45^. Reports have shown promising outcomes of ATRA, which suppressed PDAC growth by inhibiting PSCs. We also targeted macrophages with liposomal clodronate, which has been shown to be engulfed by TAMs, induce cell death, and eventually result in reduced tumor growth in vivo^46^. Activated PSCs are the main collagen-producing cells in the PDAC TME and create a dense stroma around neoplastic epithelial cells, hindering chemotherapy delivery to target cells^47^. Additionally, PSCs secrete soluble factors and/or activate cancer cell stemness signaling pathways that can induce the proliferation of cancer cells^12^. Macrophages also secrete proinflammatory cytokines to confer chemoresistance^26^. Overall, we observed a significant increase in the ability of chemotherapy to kill cancer cells when combined with stroma-depleting agents (Fig. 6).

The results of this study establish the ex vivo, PDO-based organ-chip as a reliable platform for PM in PDAC. Although microfluidic chips fabricated with polydimethylsiloxane (PDMS) have been widely used for cell culture assays involving incubation of the cells with hydrophobic drug molecules, the porous and hydrophobic nature of the material can cause quick absorption of the drug molecules. As a result, a lower drug concentration is expected to be available in the perfused medium for cellular uptake^48,49^. The lack of a control test for studying drug permeation into PDMS is a limitation of the study. However, the drug efficacy of chemotherapy with stroma depletion (group name: gemcitabine + ATRA + Clodrosome^®^) was compared with that of the chemotherapy (group name: gemcitabine)-treated group, suggesting a negligible impact of PDMS drug permeation on the final result. Moreover, due to the lower partition coefficient (log P) of gemcitabine and continuous perfusion of the new drug molecules into the chips throughout the experimental period, no substantial difference in the experimental outcome is assumed, if any^50^.

In our proof of concept study, HLA phenotyping of the organoid was not performed. In future patient-based platforms, where individualized approaches are taken (stroma cells derived from patient samples such as PBMCs), HLA mismatching would not be an issue, as all the cellular components of the chip would be developed from the same patient^7^. Future studies with a larger sample size to run a genetic analysis of the PDOs are required to confirm the tumor genetic makeup in pancreatic cancer. In addition, a personalized chip composed of immune cells such as myeloid-derived suppressor cells, lymphocytes, and dendritic cells would greatly serve to further investigate the obstacles related to treating immunologically cold PDAC^51^.

## Conclusions

In this study, we have developed a state-of-the-art multicellular tissue-chip model that shows improved long-term cell survival of primary PDOs. A limitation of this study is the use of commercially available cell lines for PSCs and monocytes rather than using patient-derived primary cells. Coculture of PDOs and stromal cells in a microfluidic chip device can recapitulate the TME of a particular patient and thus be used to evaluate the sensitivity of any anticancer drug in the laboratory prior to application in the clinic. However, we have simultaneously confirmed the profound effects of stromal cells in PDAC aggravation and the ability of cancerous epithelial cells to hijack stromal cell functions to create a favorable TME via collagen deposition and cancer-associated gene expression. Our work also provides an opportunity to expand this platform to incorporate vasculature and additional immune cells to better recapitulate the complex PDAC TME. Finally, by providing the required technical data to design a bioassay tool that can optimize drug response, we are advancing the implementation of personalized/precision medicine in PDAC.

## Materials and methods

### Microfluidic device fabrication

The two-layer microfluidic device was fabricated using a method published previously^52,53^. To create the polydimethylsiloxane (PDMS) layers, a SYLGARD 184 elastomer containing silicone precursors was used as a base along with a curing agent (Dow Chemical, Midland, MI) at a 9:1 ratio. The top layer was created by molding PDMS into a lithographically patterned SU-8 wafer. The mixed precursors were placed in a vacuum degassing chamber (Bel-Art Products, Wayne, NJ) to remove air bubbles and then placed in a 65 °C oven overnight for curing. The cured layer was detached from the mold, and individual devices were cut out. A biopsy punch (World Precision Instruments, Sarasota, FL) was used to create 0.75 mm access. A porous polyester membrane with a pore size of 0.4 µm, pore density of 4 × 10^6^ pores/cm^2^, and thickness of 10 µm (AR Brown-US, Pittsburgh, PA) was laser cut and attached to the top PDMS layer using silicon precursors. In parallel, the bottom layer was prepared by attaching a 250 µm thick silicone sheet (Rogers Corporation, Chandler, AZ) to a glass slide after cleaning both in a plasma cleaner. Following a second cleaning step in the plasma cleaner, the top layer with the membrane was bonded to the bottom layer. The resulting device had a top chamber volume of ~1 µL (length × width × height: 10 mm × 1 mm × 100 µm) and a bottom chamber volume of approximately 2.5 µL (length × width × height: 10 mm × 1 mm × 250 µm). A 100 µL media reservoir was created by cutting out 8 mm holes from the silicone sheet and attaching them to the device top. The device chambers were cleaned and sterilized with 70% isopropyl followed by a 15-min exposure under UV light before use. The device has been characterized for the absence of hypoxia and was found to pose no obstacle to drug delivery to cultured cells^52^.

### Isolation of primary cancer cells (PCCs)

Biopsy samples were collected from patients with PDAC undergoing endoscopic ultrasound at Rush University Medical Center as previously described^9^. Written consent was collected from the patients beforehand. The samples were processed immediately after the pathology laboratory confirmed a diagnosis of PDAC. An IRB-approved protocol (ORA#16071904) was used to generate the organoids. Briefly, the biopsy tissue was digested using collagenase-II, dispase-II, and DNase-I followed by several washing steps and plating with 50 µL Matrigel domes on a 24-well plate. The cells were supplemented with an organoid growth medium consisting of advanced DMEM/F12, HEPES, nicotinamide, gastrin, hEGF, A83-01, Y-27632, hFGF, and Wnt3A-R-spondin1-Noggin conditioned media (50% final volume). Organoids were formed in the 3D Matrigel within a few days of culture and were regularly supplemented and dissociated for expansion.

### Cell lines and culture

Human pancreatic stellate cells (PSCs, ScienCell Cat# 3830) were cultured in Stellate Cell Medium (SteCM, Cat# 5301); pancreatic ductal adenocarcinoma (MIA PaCa-2, ATCC Cat# CRM-CRL-1420, RRID: CVCL_0428) and monocyte (U937, ATCC^®^, CRL-1593.2^TM^, HLA phenotyped^54^) cell lines were cultured in RPMI-1640 medium (ATCC, Cat# 30-2001). All media were supplemented with 10% FBS and 1% penicillin-streptomycin and incubated in a humidified incubator at 37 °C with 5% CO_2_.

### Coculture studies and measurement of the organoid diameter

The growth of PCC organoids was measured by quantifying the diameter after seeding with either pancreatic stellate cells (PSCs) or U937 cells in the 3D Matrigel scaffold in 24-well plates. The coculture was continued for 6 days (d), and the medium from each well was replaced every other day. Imaging was performed using a Zeiss Axio Observer inverted microscope (Zeiss, Germany) on Days 1, 3, and 6 post-seeding. The diameter was calculated along the long axis of the organoid using ImageJ^®^ software (NIH, MD) calibrated against a known scale bar.

### Cell-counting kit (CCK8) assay

The proliferation of MIA PaCa-2 and U937 cells in coculture and the IC_50_ values of liposomal clodronate (Clodrosome^®^, Encapsula Nanosciences, TN) and ATRA (Sigma, MO) in U937 and PSC cells, respectively, were determined by a cell-counting kit (CCK8, Dojindo Molecular Technologies, Inc., MD) assay according to the manufacturer’s instructions. Briefly, 10 µL of WST-8 was added to the experimental cell-containing wells and incubated for up to 4 h, after which the absorbance at 450 nm was assessed using a UV microplate reader (Biotek Synergy HT, VT).

### Immunostaining, imaging, and analyses

H&E (Vector laboratories, CA, Cat# H-3502) staining was conducted according to the manufacturer’s instructions. The cells were fixed with 4% paraformaldehyde (PFA), washed twice with HBSS (Life Technologies Corporation, NY), and incubated with hematoxylin for 5 min. The cells were then incubated with a Bluing reagent for 15 s. Before and after adding eosin Y for 3 min, the cells were washed with 100% ethanol. Bright-field imaging was performed using a Zeiss Axio Observer inverted microscope.

Immunostaining of the cells cultured in wells or chips was performed after fixing the cells in prewarmed PFA and permeabilizing them with 1% Triton X-100 (Sigma). The cells were then blocked for 1 h and incubated with primary antibodies (mentioned below) overnight in a humidified chamber at 4 °C. The next day, the cells were incubated with secondary antibodies tagged with Alexa Fluor (AF) 488 or 555 for 2 h at room temperature. Confocal laser scanning microscopy (CLSM, Carl Zeiss LSM710, Germany) was employed to detect the fluorescence intensity of AF-tagged cells. Approximately ten images were captured from each sample to quantify the intensity using ImageJ^®^ software (NIH, MD). The primary antibodies used in the study were antibodies against EpCAM (Cell Signaling, Cat# 2929s), α-SMA (Abcam, Cat# ab124964), collagen-I (Invitrogen, Cat# PA5-95137), pERK (Cell Signaling, Cat# 9101L), CD68 (Cell Signaling, Cat# 76437s), cleaved caspase-3 (C.Cas-3, Cell Signaling, Cat# 9661s). The secondary antibodies were anti-mouse Alexa Flour^®^-488 (Cell Signaling, Cat# 4408s) and anti-rabbit Alexa Flour^®^-555 (Cell Signaling, Cat# 4413s). C.Cas-3 staining in the EpCAM-positive cells in the chips was performed with continuous perfusion (60 µL/h) of the reagents and diluted antibodies in the corresponding buffer. The sequence of the steps and antibody incubation time were as mentioned above.

### qPCR

Gene expression was measured after 6 days coculture of U937 cells or PSCs on transwell inserts with PCCs + PSCs or PCCs + U937 cells embedded on the bottom of the welled plate. Six-well Transwell plates were used to coculture the cells, as the small area inside the chip channel cannot accommodate the vast cell number required for the experiment. RNA was extracted from U937 cells and PSCs in coculture using an RNeasy mini kit (Qiagen, Germany). Purified RNA was then converted to cDNA using a High-Capacity cDNA Reverse Transcription Kit (Applied Biosystems, United States). qPCR was performed according to our previous protocol in a LightCycler^®^ 96 (Roche, Switzerland) using primers (Tab. S1: List of Primers)^28^. Gene expression was quantified from the ∆∆CT values normalized against the housekeeping gene GAPDH. Data are expressed as fold-changes in gene expression in cocultured cells over cells cultured alone (control). The M2/M1 ratio was calculated as the ratio of CD163/IL6 expression.

### Seeding and growth of PCCs and stromal cells in chips

Organoids were recovered from the Matrigel dome using a cell recovery solution (Corning^®^ Cell Recovery Solution, Cat# 354270, AZ). The cells were then incubated in a 37 °C water bath for 40 min, with manual shaking every 10 min to dissociate single cells mechanically. The dissociated cells were passed through a 70 µm nylon cell strainer (Corning^®^, NY). The cells were counted using a cell counter (Bio-Rad, TC20^TM^, CA) followed by the culture at 10,000 cells/well. After the organoids were fully grown on Day 7, they were collected carefully, without disrupting the morphology, and placed in an Eppendorf tube with or without PSCs and U937 cells suspended in the organoid medium. Matrigel was added to the cell suspension at a final concentration of 50%. The cell suspension was introduced into the upper chamber of the chip (sterilized) using a 10 µL pipet tip. The final cell numbers per chip were 2000 single cells from organoids grown for 7 days in welled plates, 50,000 PSCs, and 10,000 U937 cells. The dissociated single cells from the organoids failed to grow in the chips; therefore, we employed a welled plate culturing step with a known number of organoids before loading the chips. The cell-laden chips were placed in an incubator at 37 °C with 5% CO_2_ for 20 min to allow gel formation. The chips were maintained with constant perfusion of cell culture medium at 5 µL/h through the bottom channel. Both channels were separated by a 0.4 µm pore-sized polyester membrane (AR Brown-US, PA) used in commercially available standard Transwell inserts that are routinely used for cell culture. The pore size selection was a critical step, and the pore size was selected based on the size of the cells being used in the chips. Membranes with pore sizes of 3 and 8 µm are commonly used for Transwell migration assays^55^. The smaller pore-size membrane used in our chips reduced the unwanted spontaneous migration of cells into the other channel, while the delivery of cell culture medium and drugs and waste material disposal were still possible^52,56,57^. Additionally, Matrigel at a 50% dilution has an average pore size of 2 µm, which is much smaller than the cell dimension, thus allowing the Matrigel to hold cells without hindering molecule transportation^58^. The cell-laden chips were routinely observed under a microscope, and images were acquired using a Zeiss Axio Observer inverted microscope.

### IC_50_ value calculation and drug perfusion into chips

The half-maximal inhibitory concentration (IC_50_) value is a well-recognized measure of drug efficacy^59^. The cells were treated with the corresponding drug for 72 h at a concentration range before performing the viability assays. A CCK8 assay was used to determine the IC_50_ values of ATRA (3.322 µM) and Clodrosome^®^ (16.58 µg/mL) in PSCs and U937 monocytes, respectively. A Promega CellTiter-Glo^®^ 3D cell viability assay kit was used to determine the IC_50_ value of gemcitabine (33.44 nM) in the organoids. Chips treated with gemcitabine (*n* = 5) and gemcitabine + ATRA + Clodrosome^®^ (*n* = 5) were perfused with the IC_50_ values of the drugs for 72 h before fixation and imaging. Control chips (*n* = 5) were perfused with 1% DMSO. Organoids were treated similarly across the groups; the cell number and cell culture period before drug treatment remained constant for every chip. Therefore, we assume that the organoid size variability and the drug response are comparable across the groups.

### Statistical analysis

GraphPad Prism 9 (GraphPad Software, San Diego California USA, www.graphpad.com) was used to analyze the data and generate figures. Numerical results, such as organoid diameter, cell-counting kit-8 assay, invasion assay, qPCR assay, and fluorescence intensity data derived from immunofluorescence are expressed as the mean ± SEM. Images were quantified using ImageJ^®^ software (NIH, MD). Statistical analysis was performed by unpaired *t*-test. *P* values less than 0.05 were considered statistically significant and are marked with asterisks in the figures.

## Supplementary information

Supplementary Figure 1

## Data Availability

The datasets generated and/or analyzed during the current study are not publicly available for privacy reasons but are available from the corresponding author upon reasonable request.

## References

[1] Siegel RL, Miller KD, Fuchs HE, Jemal A (2021). Cancer statistics, 2021. CA Cancer J. Clin..

[2] Rahib L, Wehner MR, Matrisian LM, Nead KT (2021). Estimated projection of US cancer incidence and death to 2040. JAMA Netw. Open.

[3] Siegel RL, Miller KD, Jemal A (2020). Cancer statistics, 2020. CA Cancer J. Clin..

[4] Vincent A, Herman J, Schulick R, Hruban RH, Goggins M (2011). Pancreatic cancer. Lancet.

[5] Conroy T (2011). FOLFIRINOX versus gemcitabine for metastatic pancreatic cancer. N. Engl. J. Med..

[6] Conroy T (2018). FOLFIRINOX or gemcitabine as adjuvant therapy for pancreatic cancer. N. Engl. J. Med..

[7] Haque, M. R. et al. Organ-chip models: opportunities for precision medicine in pancreatic cancer. *Cancers*10.3390/cancers13174487 (2021). 10.3390/cancers13174487 PMC8430573 34503294

[8] Drost J, Clevers H (2018). Organoids in cancer research. Nat. Rev. Cancer.

[9] Armstrong, A. et al. Multiplex patient-based drug response assay in pancreatic ductal adenocarcinoma. *Biomedicines*10.3390/biomedicines9070705 (2021). 10.3390/biomedicines9070705 PMC8301364 34201419

[10] Thomas D, Radhakrishnan P (2019). Tumor-stromal crosstalk in pancreatic cancer and tissue fibrosis. Mol. Cancer.

[11] Neesse A (2019). Stromal biology and therapy in pancreatic cancer: ready for clinical translation?. Gut.

[12] Feig C (2012). The pancreas cancer microenvironment. Clin. Cancer Res..

[13] Yang S, Liu Q, Liao Q (2020). Tumor-associated macrophages in pancreatic ductal adenocarcinoma: origin, polarization, function, and reprogramming. Front. Cell Dev. Biol..

[14] Salnikov AV (2009). Targeting of cancer stem cell marker EpCAM by bispecific antibody EpCAMxCD3 inhibits pancreatic carcinoma. J. Cell Mol. Med..

[15] Mi K (2009). Influence of a self-assembling peptide, RADA16, compared with collagen I and Matrigel on the malignant phenotype of human breast-cancer cells in 3D cultures and in vivo. Macromol. Biosci..

[16] Cavo M (2018). A new cell-laden 3D Alginate-Matrigel hydrogel resembles human breast cancer cell malignant morphology, spread and invasion capability observed "in vivo". Sci. Rep..

[17] Collisson EA (2012). A central role for RAF->MEK->ERK signaling in the genesis of pancreatic ductal adenocarcinoma. Cancer Disco..

[18] Jayasingam SD (2019). Evaluating the polarization of tumor-associated macrophages into M1 and M2 phenotypes in human cancer tissue: technicalities and challenges in routine clinical practice. Front. Oncol..

[19] Liu J (2021). Cancer-associated fibroblasts provide a stromal niche for liver cancer organoids that confers trophic effects and therapy resistance. Cell Mol. Gastroenterol. Hepatol..

[20] Vlachogiannis G (2018). Patient-derived organoids model treatment response of metastatic gastrointestinal cancers. Science.

[21] Chen WJ (2014). Cancer-associated fibroblasts regulate the plasticity of lung cancer stemness via paracrine signalling. Nat. Commun..

[22] Ebbing EA (2019). Stromal-derived interleukin 6 drives epithelial-to-mesenchymal transition and therapy resistance in esophageal adenocarcinoma. Proc. Natl Acad. Sci. USA.

[23] Lai Benjamin, F. L. et al. Recapitulating pancreatic tumor microenvironment through synergistic use of patient organoids and organ-on-a-chip vasculature. *Adv. Funct. Mater.*10.1002/adfm.202000545 (2020). 10.1002/adfm.202000545 PMC7939064 33692660

[24] Ohlund D (2017). Distinct populations of inflammatory fibroblasts and myofibroblasts in pancreatic cancer. J. Exp. Med..

[25] Hamson EJ, Keane FM, Tholen S, Schilling O, Gorrell MD (2014). Understanding fibroblast activation protein (FAP): substrates, activities, expression and targeting for cancer therapy. Proteom. Clin. Appl..

[26] Cui R (2016). Targeting tumor-associated macrophages to combat pancreatic cancer. Oncotarget.

[27] Amit M, Gil Z (2013). Macrophages increase the resistance of pancreatic adenocarcinoma cells to gemcitabine by upregulating cytidine deaminase. Oncoimmunology.

[28] Bishehsari F (2018). KRAS mutation and epithelial-macrophage interplay in pancreatic neoplastic transformation. Int J. Cancer.

[29] Friedl P, Wolf K (2003). Tumour-cell invasion and migration: diversity and escape mechanisms. Nat. Rev. Cancer.

[30] Ozdemir BC (2014). Depletion of carcinoma-associated fibroblasts and fibrosis induces immunosuppression and accelerates pancreas cancer with reduced survival. Cancer Cell.

[31] Rhim AD (2014). Stromal elements act to restrain, rather than support, pancreatic ductal adenocarcinoma. Cancer Cell.

[32] De Wever O (2004). Tenascin-C and SF/HGF produced by myofibroblasts in vitro provide convergent pro-invasive signals to human colon cancer cells through RhoA and Rac. FASEB J..

[33] Orimo A (2005). Stromal fibroblasts present in invasive human breast carcinomas promote tumor growth and angiogenesis through elevated SDF-1/CXCL12 secretion. Cell.

[34] Gaggioli C (2007). Fibroblast-led collective invasion of carcinoma cells with differing roles for RhoGTPases in leading and following cells. Nat. Cell Biol..

[35] Goetz JG (2011). Biomechanical remodeling of the microenvironment by stromal caveolin-1 favors tumor invasion and metastasis. Cell.

[36] Labernadie A (2017). A mechanically active heterotypic E-cadherin/N-cadherin adhesion enables fibroblasts to drive cancer cell invasion. Nat. Cell Biol..

[37] Mierke CT (2008). Role of the endothelium during tumor cell metastasis: is the endothelium a barrier or a promoter for cell invasion and metastasis?. J. Biophys..

[38] Shian SG, Kao YR, Wu FY, Wu CW (2003). Inhibition of invasion and angiogenesis by zinc-chelating agent disulfiram. Mol. Pharm..

[39] Tran TA (2009). Non-anti-mitotic concentrations of taxol reduce breast cancer cell invasiveness. Biochem. Biophys. Res. Commun..

[40] Bradney MJ, Venis SM, Yang Y, Konieczny SF, Han B (2020). A biomimetic tumor model of heterogeneous invasion in pancreatic ductal adenocarcinoma. Small.

[41] Nguyen DT (2019). A biomimetic pancreatic cancer on-chip reveals endothelial ablation via ALK7 signaling. Sci. Adv..

[42] Beer M (2017). A novel microfluidic 3D platform for culturing pancreatic ductal adenocarcinoma cells: comparison with in vitro cultures and in vivo xenografts. Sci. Rep..

[43] Drifka CR, Eliceiri KW, Weber SM, Kao WJ (2013). A bioengineered heterotypic stroma-cancer microenvironment model to study pancreatic ductal adenocarcinoma. Lab Chip.

[44] Froeling FE (2011). Retinoic acid-induced pancreatic stellate cell quiescence reduces paracrine Wnt-beta-catenin signaling to slow tumor progression. Gastroenterology.

[45] Kocher HM (2020). Phase I clinical trial repurposing all-trans retinoic acid as a stromal targeting agent for pancreatic cancer. Nat. Commun..

[46] Griesmann H (2017). Pharmacological macrophage inhibition decreases metastasis formation in a genetic model of pancreatic cancer. Gut.

[47] Pandol SJ, Edderkaoui M (2015). What are the macrophages and stellate cells doing in pancreatic adenocarcinoma?. Front. Physiol..

[48] Gomez-Sjoberg R, Leyrat AA, Houseman BT, Shokat K, Quake SR (2010). Biocompatibility and reduced drug absorption of sol-gel-treated poly(dimethyl siloxane) for microfluidic cell culture applications. Anal. Chem..

[49] Wang JD, Douville NJ, Takayama S, ElSayed M (2012). Quantitative analysis of molecular absorption into PDMS microfluidic channels. Ann. Biomed. Eng..

[50] Wishart DS (2018). DrugBank 5.0: a major update to the DrugBank database for 2018. Nucleic Acids Res..

[51] Galon J, Bruni D (2019). Approaches to treat immune hot, altered and cold tumours with combination immunotherapies. Nat. Rev. Drug Disco..

[52] Wang C, Dang T, Baste J, Anil Joshi A, Bhushan A (2021). A novel standalone microfluidic device for local control of oxygen tension for intestinal-bacteria interactions. FASEB J..

[53] Wang C, Tanataweethum N, Karnik S, Bhushan A (2018). Novel microfluidic colon with an extracellular matrix membrane. ACS Biomater. Sci. Eng..

[54] Passmore JS, Lukey PT, Ress SR (2001). The human macrophage cell line U937 as an in vitro model for selective evaluation of mycobacterial antigen-specific cytotoxic T-cell function. Immunology.

[55] Justus, C. R., Leffler, N., Ruiz-Echevarria, M. & Yang, L. V. In vitro cell migration and invasion assays. *J. Vis. Exp*. 10.3791/51046 (2014). 10.3791/51046 PMC4186330 24962652

[56] Hegde M (2014). Dynamic interplay of flow and collagen stabilizes primary hepatocytes culture in a microfluidic platform. Lab Chip.

[57] Tanataweethum N (2021). Towards an insulin resistant adipose model on a chip. Cell Mol. Bioeng..

[58] Zaman MH (2006). Migration of tumor cells in 3D matrices is governed by matrix stiffness along with cell-matrix adhesion and proteolysis. Proc. Natl Acad. Sci. USA.

[59] Aykul S, Martinez-Hackert E (2016). Determination of half-maximal inhibitory concentration using biosensor-based protein interaction analysis. Anal. Biochem..

