# Supplementary figures and images for: Patient-derived pancreatic cancer-on-a-chip recapitulates the tumor microenvironment
Source: Microsyst Nanoeng. 2022 Mar 31;8:36. doi: 10.1038/s41378-022-00370-6 (PMC8971446; doi:10.1038/s41378-022-00370-6)

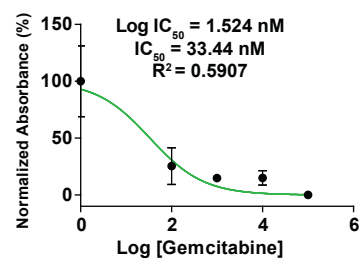

Supplement: Supplementary file 1 — Supplementary Figure 1 [file 41378_2022_370_MOESM1_ESM.pdf]
